# Effects of Dietary Modified Bazhen on Reproductive Performance, Immunity, Breast Milk Microbes, and Metabolome Characterization of Sows

**DOI:** 10.3389/fmicb.2021.758224

**Published:** 2021-11-15

**Authors:** Jian Geng, Weicheng Jin, Jingyou Hao, Mohan Huo, Yuefeng Zhang, Chunmei Xie, Baokai Zhao, Yanhua Li

**Affiliations:** ^1^College of Veterinary Medicine, Northeast Agricultural University, Harbin, China; ^2^Heilongjiang Key Laboratory for Animal Disease Control and Pharmaceutical Development, Harbin, China; ^3^Harbin Lvdasheng Animal Medicine Manufacture Co., Ltd., Harbin, China; ^4^College of Life Sciences, Northeast Agricultural University, Harbin, China; ^5^Liaoning VICA Agriculture and Animal Husbandry Ecological Food Co., Ltd., Xincheng, China; ^6^Harbin Herb & Herd Bio-Technology Co., Ltd., Harbin, China

**Keywords:** modified Bazhen powder, reproductive performance, milk microbes, metabolome characterization, sow

## Abstract

Bazhen is a classic prescription used for the prevention of qi and blood deficiency. The present study aimed to investigate the effects of dietary supplementation with modified Bazhen powder (MBP) on sows during lactation. Forty pure-bred Yorkshire sows on day 100 of gestation were randomly fed a standard diet supplemented with 20 g MBP per sow per day (MBP group) or without (control group) during -14 to 7 days relative to parturition. Results showed that the serum levels of interleukin 2 (IL-2), immunoglobulin A (IgA), and IgG were higher, whereas IL-10 level was lower in sows fed with MBP diet than in controls on day 7 postpartum. A significantly elevated proportion of serum CD4^+^ T cells and a slight increase in the ratio of CD4^+^ to CD8^+^ T cells in the MBP group were also observed. Furthermore, MBP supplementation improved gastrointestinal function of postpartum sows, evidenced by increased levels of motilin, gastrin, and nitric oxide. Ultra high-performance liquid chromatography combined with a quadrupole time of flight and tandem mass spectrometer identified a total of 21 absorbed milk components. 16S rRNA gene amplicon sequencing data revealed that the microbiota diversity of the colostrum and transitional milk in the MBP group was increased. At the genus level, relative abundances of *Enterococcus* and *Anaerostipes* were significantly lower in the MBP group on day 0 of lactation. Metabolomic analysis showed that 38 metabolites were upregulated, and 41 metabolites were downregulated in the transitional milk; 31 metabolites were upregulated and 8 metabolites were downregulated in the colostrum in response to MBP. Metabolic pathways, protein digestion and absorption, and biosynthesis of amino acids were enriched in the colostrum and transitional milk. Our findings provide new insights into the beneficial effects of MBP, highlighted by the changes to the microbiota and metabolomic profile of breast milk from sows fed with an MBP-supplemented diet. Thus, MBP should be considered as a potential dietary supplement for lactating sows in pork production.

## Introduction

Normal physiological states of perinatal sow are essential to maintain its production at an optimum level (Heuß et al., 2019). In particular, the amount and quality of milk during late gestation and lactation are found to influence the survival capacities, health, and growth of piglets (Devillers et al., 2011; Ferrari et al., 2014). Sow’s milk is a complex biological fluid produced by the mammary glands and is the main food for piglets during their initial stages of development. Breast milk contains the necessary macronutrients, micronutrients, minerals vitamins, hormones, and other bioactive molecules that promote the growth and immune defense of piglets (Ballard and Morrow, 2013; Scano et al., 2016). However, in some cases, a reduction in breast milk secretion of sow can occur after birth caused by several factors including lack of breast stimulation, natural environment, and nutritional states. This can cause serious problems with piglet growth and survival. Currently, in intensive pig farms, the sows are kept in individual housing systems that restrict the movement of sows, causing stress, hypoimmunity, and a reduction in milk production. Production efficiency influences the supply of market hogs and, consequently, pork. Thus, increasing the production of breast milk and the content of microcomponents that have beneficial effects on health maintenance and immune defense is of great significance to pig breeding.

There are many methods to improve the quality and quantity of breast milk, such as feeding a well-balanced diet, drinking more water, and stimulating the breasts. In addition, there are natural ingredients that can be added to the diet to promote the healthy production of sow’s milk. Traditional Chinese medicine (TCM), originating thousands of years ago, has been used to treat illnesses and maintain human health. Chinese herbal medicine (CHM) contains bioactive components that possess anti-inflammatory, antibacterial, antioxidation, and immune-enhancing properties. More recently, CHM has been reported to enhance lactation performance in sows, chickens, and other animals. Hordei Fructus Germinatus can effectively enhance lactation in rats by influencing the prolactin (PRL)/JAK-STAT signaling pathway (Zhang et al., 2021). Dietary rumen-protected betaine supplementation improves lactation performance of dairy cows fed with diets moderately deficient in methionine (Wang et al., 2020). A fermented CHM mixture consisting of 18 herbal supplements improves milk performance and immune function in dairy cows under heat stress conditions (Shan et al., 2018). Because of the natural origin, CHM will not cause excessive drug buildup or toxicity, and CHM can be considered as a safe and suitable substitute for antibiotics in animal feeding.

To date, there has been little information about the function of CHM mixture on the reproductive performance, immunity, milk microbes, and metabolic profile of sows. Bazhen, a traditional Chinese herbal prescription containing eight commonly used herbs, prevents qi and blood deficiency. Qi and blood are two essential concepts in Chinese medicine. Qi and blood deficiency translates loosely into a lack of energy and occurs when the body or a particular organ is insufficiently nourished. Bazhen has also shown many health benefits, such as alleviating anemia and asthenia, as well as increasing appetite (Song et al., 2014). Furthermore, modified Bazhen has been reported to modulate immunity and protect nerves (Lee et al., 2014). In a clinical study, Jiawei Bazhen decoction was proven to be safe and reliable for a mother and fetus and can help reduce the rate of cesarean section playing a positive role in promoting natural delivery (Ke et al., 2015). In addition, modified Bazhen can dramatically prevent the apoptosis of oocyte and granulosa cells (Liu et al., 2019). Sijunzi decoction, which is a part of Bazhen, has been found to increase the expression of PRL in rats. Based on the multiple benefits of Bazhen for females, we hypothesized that modified Bazhen powder (MBP) may have a positive impact on the productivity and postpartum state of sows. Here we showed that dietary MBP supplementation improved the reproductive performance, serum traits, and immune status of sows, as well as changing breast milk microbes and metabolome characterization. MBP should be considered as a potential functional feed additive for sows. Given the biological similarity between pigs and humans, our study may offer a scientific nutritional reference for perinatal mothers.

## Materials and Methods

### Animals

The animal experiments were carried out at Weijia great grandparent farm in Liaoning province of China (March 2020 to January 2021). There were 2,400 purebred Yorkshire sows in the herd with a 3-week batch farrowing system, which ensured that all experimental sows have a due date of the same day. Animal protocols were used throughout the research under the approval of the Experimental Animal Research Ethics Committee of Northeast Agricultural University (SRM-11). Animals suffered from no obvious stress in the whole experimental process. Participants were blinded to group assignments.

### Modified Bazhen Powder Formula

A traditional nourishing MBP formula was adopted based on traditional Chinese pharmacopoeia. MBP formula used contained 15% of Astragalus root (*Astragalus membranaceus*), 15% of Atractylodes rhizome (*Atractylodes lancea*), 15% of Hoelen (*Poria cocos Wolf*), 11.25% of Glycyrrhizae Radix (*Glycyrrihiza uralensis Fischer et DC*.), 11.25% of Raix Rubra (*Paeonia albiflora Pallas var. trichocarpa Bunge*), 10% of Angelica root (*Angelica sinensis*), 10% of Rehmanniae Radix et Rhizoma (*Rehmannia glutinosa Liboschitz*), 7.5% of Ziziphus jujuba Mill (*Ziziphus zizyphus*) and 5% of Ligustici Chuanxiong Rhizoma (*Ligusticum chuanxiong Hortorum*). Herbs were purchased from an authentic herb supplier in the local market of Harbin.

### Animals, Diets, and Experimental Design

Forty pure-bred Yorkshire sows with three parities were studied during gestation and lactation, that is, -14 to 7 days relative to parturition. Sows in the control group (control diet, *n* = 20) were fed a standard gestation and lactation diet, and sows in the MBP group (MBP diet, *n* = 20) were fed the same diet supplemented with 20 g of MBP mixture per sow per day (10 g twice a day) throughout the experiment. Each sow was offered 2.8 kg of a standard feed, which was given twice daily.

Feed diet was provided by the Weijia Biology Company (Beijing, China). Supplementary Table 1 lists the ingredients and compositions of the gestation and lactation diet. The MBP mixture was supplemented daily by top-dress feeding into the diet for individual sows.

The same growing environment was shared by all sows, where relative humidity and temperature were controlled automatically. All sows were kept in individual gestation stalls (2.3 × 0.65 m) from days 100 to 110 of gestation. On the 110th day of pregnancy, sows were moved into the farrowing crates. Hereafter, individual sow farrowing stalls (2.2 × 1.4 m) were used to house sows, and they had an enclosed heated creep area for piglets.

### Sample Collection

Milk samples were collected from sows on days 0 and 7 after parturition. Colostrum samples were collected within 3 h after the birth of piglets. Transitional milk samples were collected between 8:00 and 11:00 AM on day 7 after parturition. After collection, the colostrum and milk samples were immediately placed in liquid nitrogen for further analysis.

On days 0 and 7 after parturition, we collected blood samples from ear marginal veins and grouped them in two parts: one collected into Vacutainer collection tubes containing anticoagulants and transported to the laboratory for analysis immediately, and the other frozen in liquid nitrogen for further analysis after 15 min of centrifugation at 3,000*g* at 4°C.

### Physiological and Biochemical Parameter Analysis

Through the commercial enzyme-linked immunosorbent assay (ELISA) kits (Beijing Sino-uk Institute of Biological Technology, Beijing, China), the levels of PRL, progesterone, estradiol, immunoglobulin A (IgA), IgG, IgM, interleukin 10 (IL-10), IL-2, interferon γ, tumor necrosis factor α (TNF-α), nitric oxide (NO), gastrin, and motilin in the blood or serum samples were measured under the manufacturer’s instructions.

### Flow Cytometry Assay

Flow cytometry (Bricyte E6, Shenzhen, China) was used to quantify T-cell subsets in peripheral blood to analyze sow’s immunity. Using fluorochrome-conjugated monoclonal antibodies (Southernbiotech, United States) for CD4^+^/CD3^+^ and CD8^+^/CD3^+^ T cells, two-color phenotyping was used for immunophenotyping of lymphocytes. On the same day, we process these samples and tested them within 2 h. The CD4^+^/CD8^+^ ratio was calculated to reflect immune system health.

### Determination of the E-C3bR and E-Icb Rosette Ratios

Erythrocyte immunity was estimated by determining E-IC and E-C3bR rosettes. Their rosette ratios were determined following a previous study (Guan et al., 2014). The rosette ratios of E-C3bR were calculated using the formula: the rosette ratios of E-C3bR (%) = N1/200 × 100%, where N1 represents the number of erythrocytes binding to one or more sensitized yeast cells. The rosette ratios of E-IC were calculated using the formula: the rosette ratios of E-IC (%) = N2/200 × 100%, where N2 represents the number of eryth-rocytes binding one or more unsensitized yeast cells.

### Milk Parameter Analysis

Commercial ELISA kits were used to measure the activity level of insulinlike growth factor (IGF) and epidermal growth factor (EGF) activity in the milk of sows according to the manufacturer’s instructions (Beijing Sino-uk Institute of Biological Technology, Beijing, China). The contents of fat, protein, lactose, and dry matter in the sow’s milk were detected by an infrared spectroscopy method using CombiFoss 6000 (Foss Electric, Hillerød, Denmark).

### Traditional Chinese Medicine Ingredient Analysis

For characterizing ingredients of the transitional milk after MBP administration, we used ultrahigh-performance liquid chromatography combined with a quadrupole time of flight and tandem mass spectrometer (UHPLC-Q-TOF-MS/MS) to analyze the milk constituents. A Shimadzu Nexera UHPLC LC-30A system with a Waters UPLC BEH C_18_ column (2.1 × 100 mm, 1.7 μm) was applied to LC-MS/MS analysis. Sample volume (5 μL) was separated with a flow rate of 0.4 mL/min. Moreover, 0.1% formic acid in water (A) and in acetonitrile (B) constituted the mobile phase. The multistep linear elution gradient conditions were stated as follows: 0 to 3.5 min, 95% to 85% A; 3.5 to 6 min, 85% to 70% A; 6 to 6.5 min, 70% to 70% A; 6.5 to 12 min, 70% to 30% A; 12 to 12.5 min, 30% to 30% A; 12.5 to 18 min, 30% to 0% A; 18 to 22 min, 0% to 0% A. In the IDA acquisition mode, Analyst TF 1.7 software and an AB SCIEX Triple TOF 5600 mass spectrometer were used to collect MS/MS data. The settings of ESI heater temperature, pressures of nebulizer gas, auxiliary gas and curtain gas were 550°C, 55 psi, 55 psi and 35 psi. Besides, Ion Spray Voltage Floating was set to 5500 V in positive ion mode and −4000 V in negative mode respectively.

### Metabolite Analysis

Milk samples were extracted with 80% cold methanol. Metabolites were analyzed by UPLC-Q-TOF-MS/MS (UPL; Shimadzu, Japan) with an ACQUITY UPLC HILIC column (100 × 2.1 mm, 1.8 μm, Waters, United Kingdom). The sample injection volume was set at 2 μL, and the flow rate was set at 0.4 mL/min. The solvent system was composed of 0.1% formic acid in water (A) and in acetonitrile (B). Gradient elution was as follows: 95:5 V/V at 0 min, 10:90 V/V at 10.0 min, 10:90 V/V at 11.0 min, 95:5 V/V at 11.1 min, and 95:5 V/V at 14.0 min. The ESI source operation parameters included ion source gas I (GSI) of 55 psi, gas II (GSII) of 60 psi, curtain gas (CUR) of 25.0 psi, ion spray voltage (IS) of 5,500 V (positive) and -4,500 V (negative), and source temperature of 500°C. For determining the trends in metabolic profile intragroup, statistics function prcomp within R was applied to unsupervised principal component analysis (PCA). We used absolute Log2FC (fold change) ≥ 1 and VIP ≥ 1 to determine significant regulation of metabolites between groups. OPLS-DA (orthogonal partial least-squares discriminant analysis) result was taken as a basis to extract VIP values. The interaction pathways and the possible biological roles of potential biomarkers were analyzed and visualized by the MetaboAnalyst database and KEGG database.

### 16S Ribosomal RNA Amplicon Sequencing of Sow’s Milk

The genomic DNA from the milk samples was extracted using CTAB method. The agarose gel electrophoresis was used for estimating the purity and concentration of DNA. The V4 region of the 16S rRNA genes was amplified using specific primer (515F-806R) with the barcode. Polymerase chain reaction (PCR) amplification was performed with the Phusion^®^ High-Fidelity PCR Master Mix with GC Buffer kit (New England Biolabs). The amplification procedure was as follows: initial denaturation at 98°C for 1 min, followed by 30 cycles of denaturation at 98°C for 10 s, annealing at 50°C for 30 s, and elongation at 72°C for 30 s and, finally, 72°C for 5 min. Next, The PCR products of the same sample were mixed with 1 × loading buffer and detected by 2% agarose gel electrophoresis. Then, mixture PCR products were purified by a Gel Extraction Kit (Qiagen, Hilden, Germany). Following the manufacturer’s instructions, we built a gene library with the TruSeq^®^ DNA PCR-Free Sample Preparation Kit (Illumina, United States) and added index codes. The Agilent Bioanalyzer 2100 system and the Qubit 2.0 Fluorometer (Thermo Scientific, United States) were used to evaluate the library quality after amplification. Purified amplicon samples were pooled to be equimolar and paired-end sequenced (2 × 250 bp) on an Illumina MiSeq platform.

### Statistical Analysis

Statistical differences of two groups were compared using the Student *t* test (GraphPad Prism 8.0 software; GraphPad Software 250 Inc., La Jolla, CA). Differences were at a significant level when *p* < 0.05. How metabolites correlated with milk bacterial species were estimated by Spearman correlation coefficients. For determining significant differences in metabolites, we performed Wilcoxon rank sum tests.

## Results

### Effects of Modified Bazhen Powder on Sow’s Performance

Table 1 details information on the sows included in this study. Supplementation of MBP to the sow diets had no effect on the total number of piglets born, piglets born alive, piglets stillborn and the survival rate of piglets. However, MBP diets significantly increased the number of piglets born healthy and average milk yield, but significantly decreased total labor course and farrowing interval of sows.

**TABLE 1:** Sow performance during lactation fed a standard diet without or with MBP.

	Control	MBP	SEM	*p* value
Total born	14.25	15.45	0.88	0.18
Born alive	12.35	13.5	0.7	0.08
Stillborn	1.85	1.75	0.31	0.75
Healthy born	12.1	13.6*	0.66	0.02
Piglets birth weight (kg)	1.36	1.43	0.66	0.33
Survival rate (%)	90.9	92.6	0.03	0.51
Total labor course (min)	241.9	218.4*	11.56	0.049
Farrowing interval	17.64	14.44*	1.2	0.01
Average milk yield (kg/d)	5.77	6.96*	0.54	0.03

### Effects of Modified Bazhen Powder on the Immunity of Sows

**FIGURE 1:**
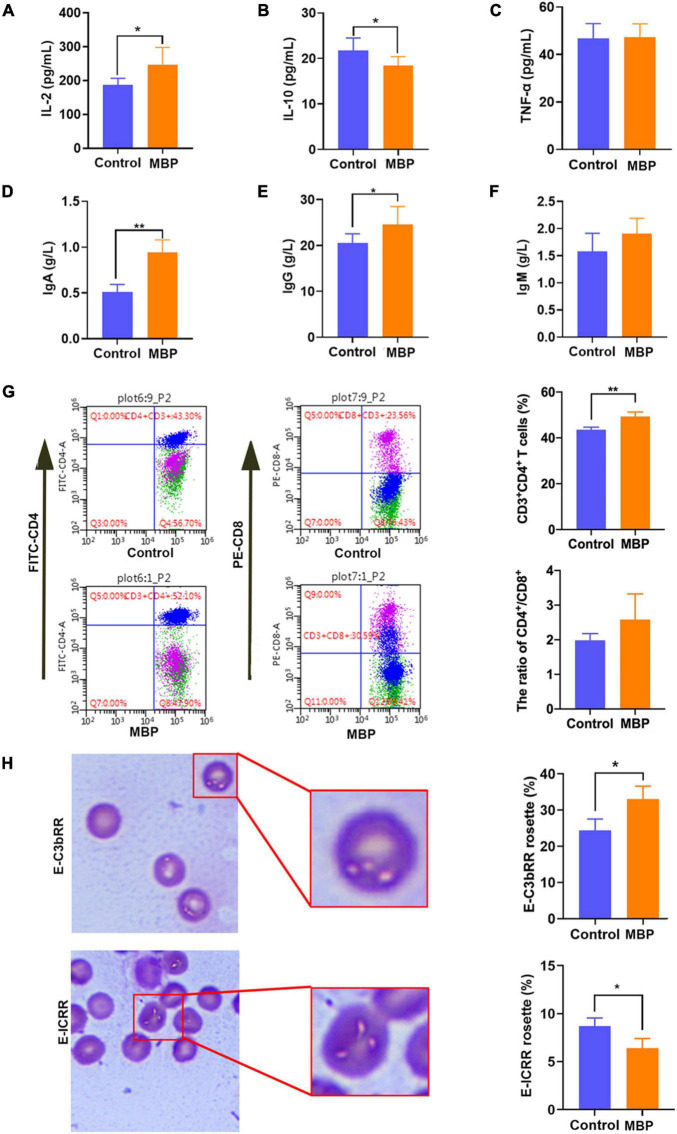
Effects of MBP on the immunity of sows on day 7 postpartum. **(A–C)** Concentration of IL-2, IL-10, and TNF-α in the serum of sows. **(D–F)** Concentration of immunoglobulins IgA, IgG, and IgM in the serum of sows. **(G)** T lymphocyte subsets in peripheral blood detected by flow cytometry. **(H)** Differences in E-C3bRR and E-IC rosette ratios were discerned for comparing of erythrocyte immune function. Data represent mean ± SD. *p < 0.05, **p < 0.01 versus control.

To investigate the effects of MBP on the modulation of immunity, crucial serum cytokines including IL-2, IL-10, and TNF-α were determined. As shown in Figures 1A–C, MBP administration significantly increased the serum level of IL-2, but significantly decreased IL-10 compared with the control sows. However, the level of serum TNF-α did not differ between the different groups.

Furthermore, serum immunoglobulins (IgA, IgG, IgM) were detected. Significantly elevated levels of the serum IgA and IgG were observed in MBP-fed sows (Figures 1D,E). Serum IgM level tended to be increased, although the difference was not statistically significant (Figure 1F).

We next assessed immune activation by incorporating both the proportion of CD4^+^ T cells and the ratio of CD4^+^/CD8^+^. A significantly higher proportion of CD4^+^ T cells and a slight increase in the ratio of CD4^+^/CD8^+^ were observed in the MBP-fed sows (Figure 1G).

As shown in Figure 1H, the E-ICRR was significantly increased, whereas the E-C3bRR was markedly decreased in the MBP group compared with the control group, indicating that MBP improved the innate immune function of erythrocytes.

### Effects of Modified Bazhen Powder on the Levels of Hemoglobin and Important Serum Hormones in Sows

**FIGURE 2:**
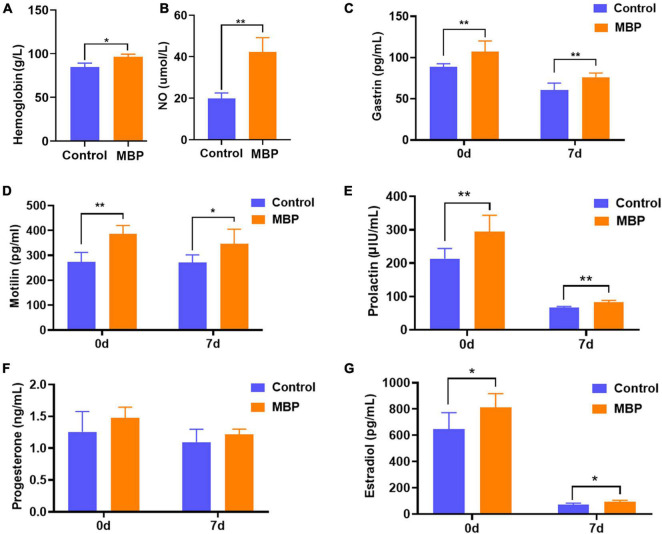
Effects of MBP on physiological and biochemical parameters in the blood of lactating sows. **(A,B)** Levels of hemoglobin and serum NO in sows on the day of farrowing. **(C,D)** Levels of serum gastrin and plasma motilin of sows on days 0 and 7 postpartum. **(E–G)** Serum levels of PRL, progesterone, and estradiol of sows on days 0 and 7 postpartum. Data represent mean ± SD. *p < 0.05, **p < 0.01 versus control.

Changes in blood traits were measured to further explore the effects of MBP on lactating sows. As shown in Figures 2A,B, dietary supplementation with MBP significantly increased the concentrations of hemoglobin and serum NO on day 7 of lactation. In addition, the concentrations of two important hormones gastrin and motilin related to the gastrointestinal system were significantly higher in the MBP group compared with control on days 0 and 7 postpartum (Figures 2C,D). Furthermore, serum levels of PRL and estradiol were significantly increased in sows on days 0 and 7 postpartum (Figures 2E,G). Although not statistically significant, there was an increasing trend of progesterone content in sows with the MBP diet (Figure 2F).

### Effects of Modified Bazhen Powder on Milk Performance of Lactating Sows

**FIGURE 3:**
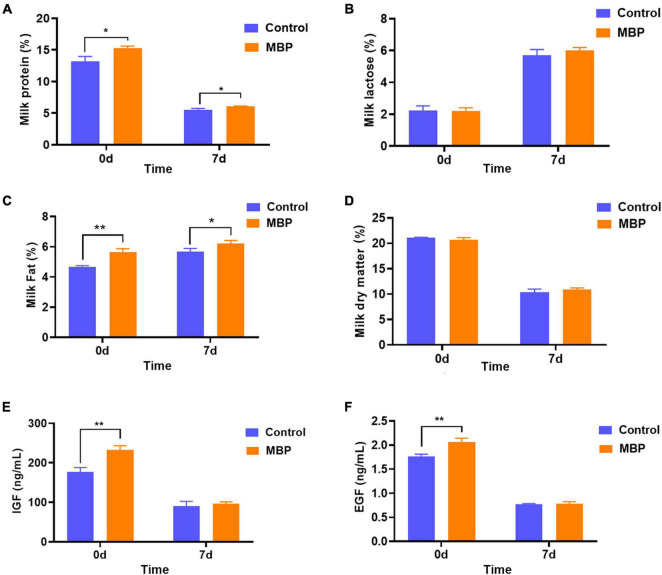
Effects of MBP on nutritional composition and growth factors in the colostrum and transitional milk (day 7) of sows. **(A)** Protein content. **(B)** Lactose content. **(C)** Fat content. **(D)** Dry matter content. **(E)** Concentration of IGF. **(F)** Concentration of EGF. Data represent mean ± SD. *p < 0.05, **p < 0.01 versus control.

Milk composition was analyzed on days 0 and 7 after parturition. Results showed that MBP supplementation significantly increased the content of protein and fat, although it did not significantly alter the content of lactose and the dry matter in the colostrum and transitional milk (Figures 3A–D).

IGF and EGF are two of the major milk-derived peptide growth factors found in milk during sow lactation. As shown in Figures 3E,F, the concentrations of IGF and EGF were higher in the colostrum from MBP-fed sows compared with the control. The concentrations of IGF and EGF in the day 7 milk did not differ between the two groups (Figures 3E,F).

### Identification of Milk Constituents

**FIGURE 4:**
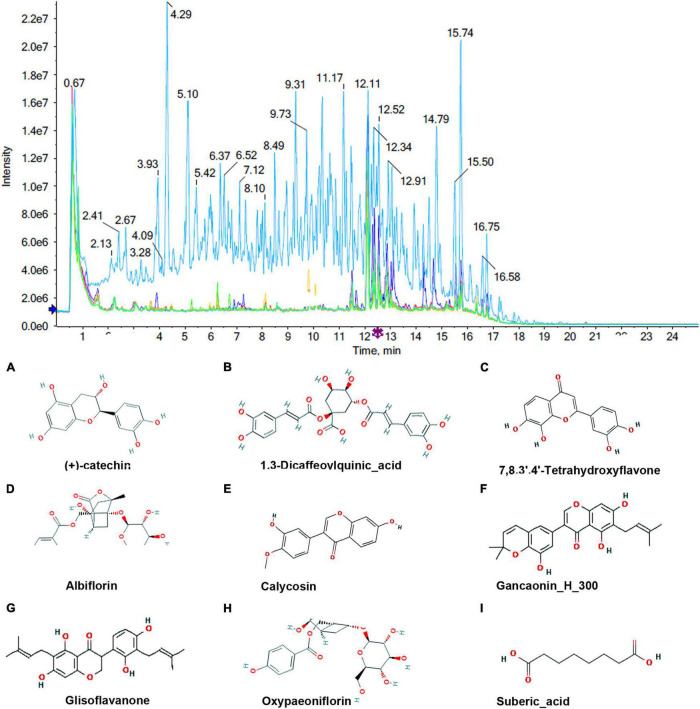
Chromatograms and chemical structures of MBP metabolites in the transitional milk of sows after dietary supplementation of MBP. The chromatograms in negative mode. **(A–I)** Absorbed milk components (+)-catechin, 1.3-Dicaffeovlquinic_acid 7,8.3.4-Tetrahydroxyflavone, Albiflorin, Calycosin, Gancaonin, Glisoflavanone, Oxypaeoniflorin and Suberic_acid.

**FIGURE 5:**
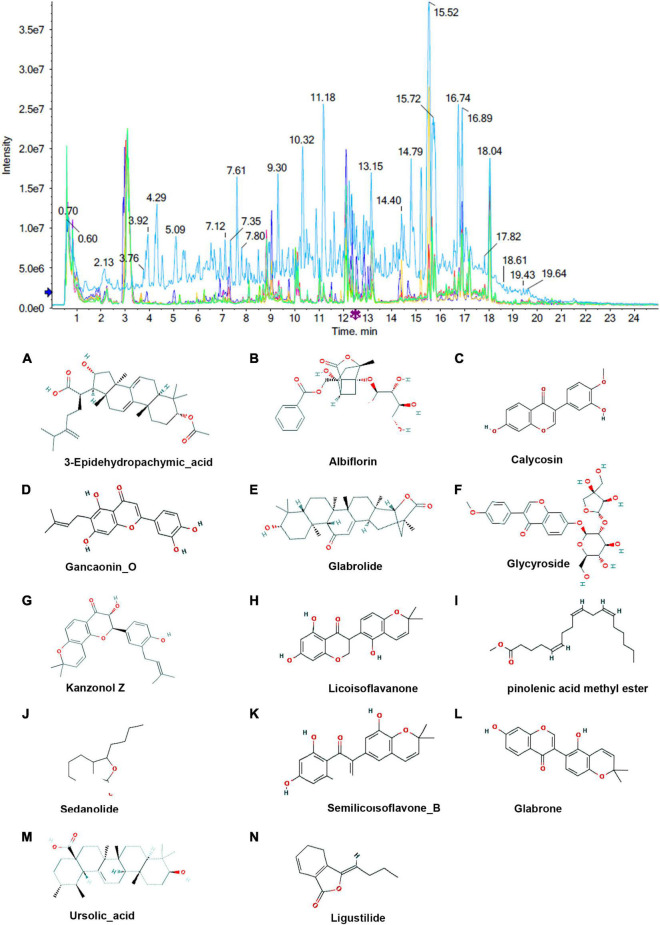
Chromatograms and chemical structures of MBP metabolites in the transitional milk of sows after dietary supplementation of MBP. The chromatograms in positive mode. **(A–N)** Absorbed milk components 3-Epidehydropachymic_acid, Albiflorin, Calycosin, Gancaonino, Glabrolide, Glycyroside, Kanzonol z, Licoisoflavanone, pinolenic acid methyl ester, Sedanolide, Semilicoisoflavone_B, Glabrone, Ursolic_acid and Ligustilide.

To identify the absorbed milk components, the total ion current (TIC) chromatograms of milk samples from the control group and the MBP group were obtained by UHPLC-MS. As shown in Figures 4, 5, a total of 21 compounds were tentatively characterized in negative ion mode and positive ion mode.

### Effects of Modified Bazhen Powder on Milk Microbiota Composition

**FIGURE 6:**
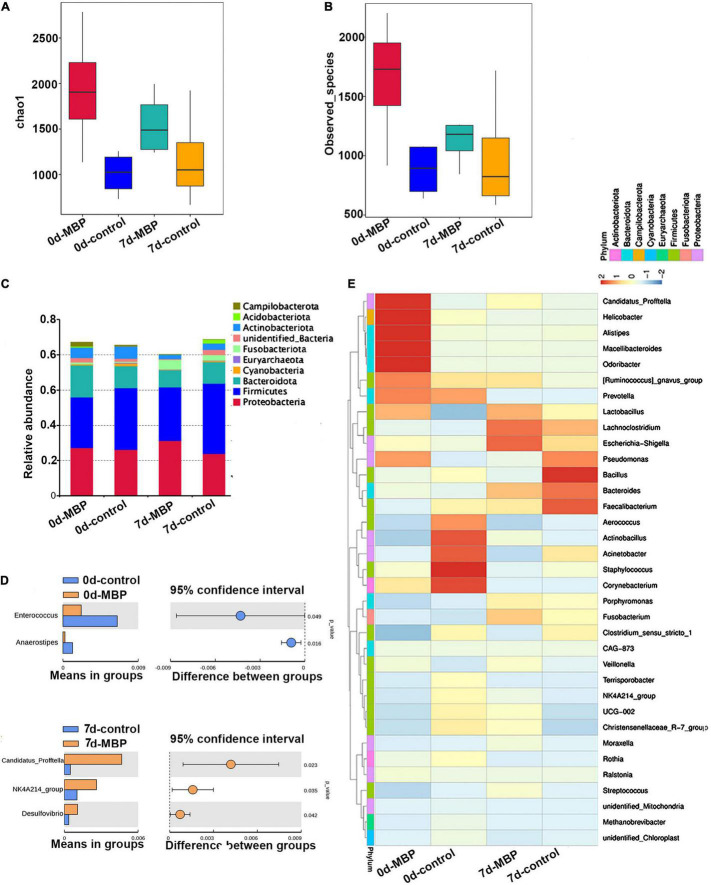
Predominant bacteria in milk samples from sows fed with the MBP diet. Microbial α-diversity assessed by **(A)** Chao1 richness estimator and **(B)** observed species were calculated. **(C)** Phylum level. **(D)** Significant differences in bacteria at the genus level in milk samples between the two groups. **(E)** Relative abundance of bacterial taxa at the genus level (top of 35).

Bacterial taxonomic composition in sows’ milk was detected by PCR amplification and pyrosequencing of 16S rRNA. The milk microbiota alpha diversity was estimated by observed_species and Chao1 index. We observed that the alpha diversity of microbiota tended to increase after receiving MBP diet on days 0 and 7 of lactation (Figures 6A,B). In addition, lactation time may influence the observed_species and Chao1 index in the MBP group. The top 10 phyla in the different groups are shown in Figure 6C. The relative contribution of the most dominant phyla (Proteobacteria and Firmicutes) to the overall composition was similar for the control and MBP groups on days 0 and 7 of lactation. Interestingly, the microbiota of the milk samples in the MBP group had a slight increase in the relative abundance of Proteobacteria and a decrease in Firmicutes especially on day 7 of lactation (Figure 6C). At the genus level, relative abundances of Enterococcus and Anaerostipes were significantly lower in the MBP group on day 0 of lactation (Figure 6D). The genus level of *Candidatus profftella*, NK4A214_group, and *Desulfovibrio* was significantly increased in the milk of MBP group on day 7 of lactation (Figure 6D).

Furthermore, the relative bacterial community abundance at the genus level in the heat map was observed. On day 0 of lactation, the proportions of five predominant taxa including *C. profftella*, *Helicobacter*, *Alistipes*, *Macellibacteroides*, and *Odoribacter* in the MBP milk increased, whereas the relative abundance *Aerococcus*, *Actinobacillus*, *Acinetobacter*, *Staphylococcus*, and *Corynebacterium* were decreased compared with control. On day 7 of lactation, the dominant genera in the control group were *Pseudomonas*, *Bacillus*, *Bacteroides*, and *Faecalibacterium*. The dominant genera in the MBP group were *Lactobacillus*, *Lachnoclostridium*, and *Escherichia*-*Shigella* (Figure 6E).

### Effects of Modified Bazhen Powder on Metabolite Profiling of Sow’s Milk

A PCA approach was used to identify the difference in the spectral composition of milk in the two groups. As suggested by OPLS-DA models, obvious separations existed between the control (green dots) and MBP (red dots) groups on both 0 (*R*^2^*X* = 0.396, *R*^2^*Y* = 0.99, *Q*^2^ = 0.562) and 7 days (*R*^2^*X* = 0.511, *R*^2^*Y* = 0.996, *Q*^2^ = 0.781) of lactation (Supplementary Figures 1A,B). On day 0, there were 39 differential metabolites (DFMs) between MBP and control, of which 31 DFMs were upregulated and 8 DFMs decreased in the MBP. On day 7, there were 79 DFMs between MBP and control, of which 38 DFMs were upregulated and 41 DFMs decreased in the MBP (Supplementary Figure 1C). All identified DFMs from the milk samples between the two groups are shown in Supplementary Table 2.

**FIGURE 7:**
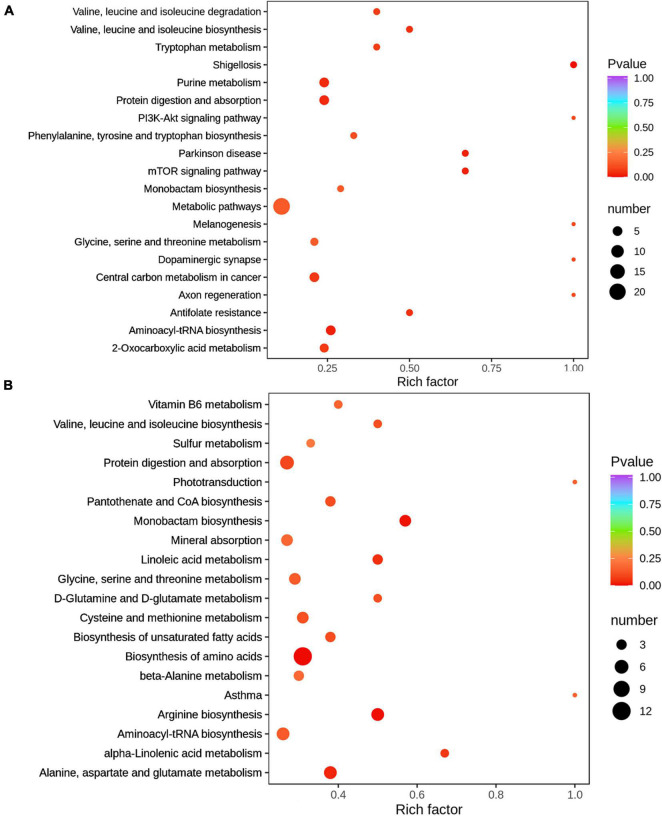
Milk metabolomic pathways analysis of sows fed with the MBP diet. **(A)** Top 20 of pathway enrichment in the colostrum of sows treated with MBP diet. **(B)** Top 20 of pathway enrichment in the transitional milk of sows treated with MBP diet. The *x* axis indicates the rich factor corresponding to each pathway, and the *y* axis indicates name of the KEGG metabolic pathways. The size and color of bubbles represent the number and degree of enrichment of different metabolites, respectively.

The metabolome map revealed main pathways on day 0 included aminoacyl-tRNA biosynthesis, metabolic pathways, protein digestion and absorption, purine metabolism, and center carbon metabolism in cancer, whereas the main pathways on day 7 included protein digestion and absorption, center carbon metabolism in cancer, arginine biosynthesis, alanine, aspartate and glutamate metabolism, biosynthesis of amino acids, monobactam biosynthesis and glycine, and aminoacyl-tRNA biosynthesis, based on the milk DFMs between the MBP and control groups (Figure 7).

### Correlation Analysis of Milk Microbiota and Metabolomics

**FIGURE 8:**
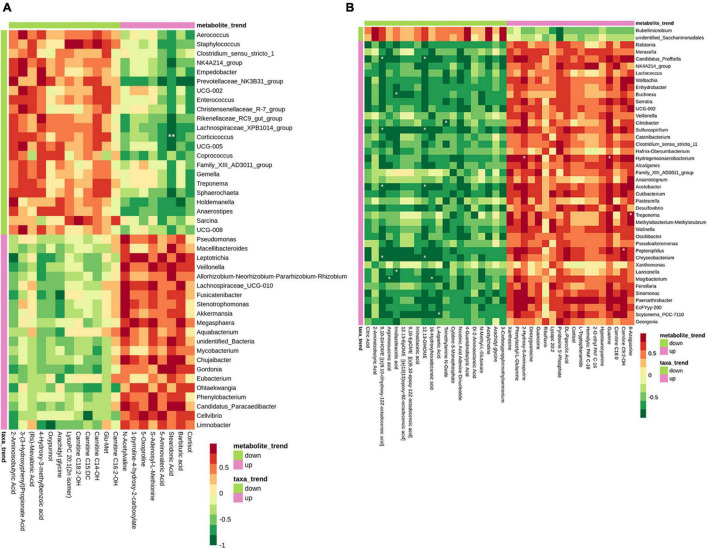
Correlation analyses between bacterial genera and metabolites in the sow’s milk. Heat map correlation showing the associations among bacterial genera and metabolite in the colostrum **(A)** and transitional milk **(B)**. *p* Values for pairwise comparisons of metabolites and bacterial genera. *p* Values are shown as *p < 0.05, **p < 0.01.

To determine the potential relationship between the changes of microbiota and the metabolites in the sow’s milk, we analyzed the correlation between the DFMs and the microbiota by calculating spearman correlation coefficients. On day 0 of lactation, *Corticicoccus* was negatively associated with stearidonic acid at the genus level (Figure 8A). On day 7 of lactation, *Hydrogenoanaerobacterium* was positively correlated guanine and 2-hydroxy-6-aminopurine. *C. profftella*, *Sulfurospirillum*, *Acetobacter*, and *Chryseobacterium* were negatively correlated 12,13-DiHOME. *C. profftella*, *Sulfurospirillum*, *Acetobacter*, *Chryseobacterium* were negatively correlated 9,10-DiHOME [(±)9,10-dihydroxy-12Z-octadecenoic acid] (Figure 8B).

## Discussion

**FIGURE 9:**
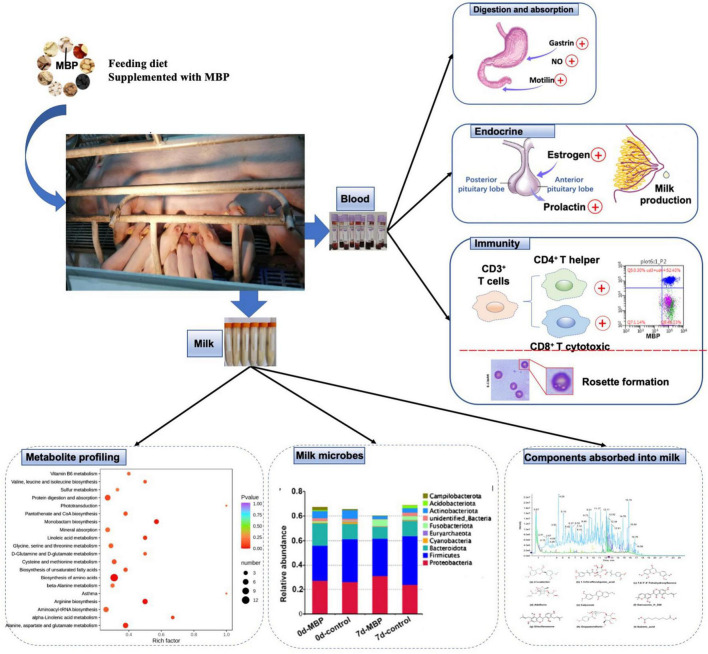
Diagram of the mechanism of MBP’s effect on postpartum sows.

Animal feed is critical to the livestock industry; however, the application of antibiotics in animal diets can cause drug residues, which will threaten human health. Traditional CHM is considered as a strategy of replacing some antibiotics and to increase animal production efficiency and profitability. Because of differences in biosynthesis and metabolic pathways, the CHM metabolites exhibit significantly different chemical compositions and clinical efficacy. In the present study, feed diet containing a traditional MBP mixture improved reproductive performance, serum traits, and immunity of sows (Figure 9). These results indicated our MBP to be a potential feed additive for lactating sows. In addition, our results revealed the differences of microbiota, metabolome, and pathways in the milk between the control and MBP groups, which might determine the co-occurrence network in the milk functions by the supplementation of MBP in sows.

MBP diets improved sow’s energy usage and performance evidenced by the increased number of piglets born healthy and average milk yield, while significantly decreased farrowing interval of sows. These benefits contribute to pork production. The MBP-supplemented diets also improved the postpartum immunity of sows. First, we detected the decreased serum levels of IL-10 and increased IL-2, IgA, and IgG in sows with MBP diet. IgG and IgA in the sow’s milk on day 7 postpartum were detected to assess the transfer of passive immunity (Gerber et al., 2012). Tan et al. have reported that dietary supplementation of *Astragalus* polysaccharides, which is a CHM, enhanced the presence of IgG and IgM in 0-h colostrum of sows (Tan et al., 2017). Another laboratory showed that feeding with garcinol significantly increased IgG and IgA in the colostrum and milk of sows (Wang et al., 2019). Furthermore, Bazhen supplement in chicken increases serum IgG and γ-globulin levels (Lien et al., 2013), and modified Bazhen can modulate immunity and protect nerves (Lee et al., 2014). Our results were consistent with these previous studies and demonstrated the ability of MBP supplementation to promote immunoglobulin synthesis. Besides, it is known that IL-10 is both an anti-inflammatory cytokine and an immunosuppressive cytokine, which inhibits numerous T-cell and antigen-presenting cell functions (Bromberg, 1995). IL-2 is identified as a T-cell growth factor, and it can quickly induce an innate immune response. The change trend of the levels of IL-2 and IL-10 in sows was in line with the expectation of immune enhancement by MBP. Furthermore, the main cellular source of IL-2 was activated CD4^+^ T cells. From the current study, the dramatically elevated proportion of serum CD4^+^ T cells and a slight increase of the ratio of CD4^+^ to CD8^+^ T cells in the MBP group were observed at the end of the experiment. Both helper/inducer (CD4^+^) T cells and suppressor/cytotoxic (CD8^+^) T cells are the main T lymphocyte subsets. CD4^+^ T cells are considered to maintain an active immune response, whereas CD8^+^ T cells are responsible for defending against intracellular pathogens (Qu et al., 2015). In general, an elevation of the blood CD4^+^/CD8^+^ ratio is an important indicator of a strong immune system. Thus, our findings of a change in the T lymphocyte subsets involved in the immune response suggest the improvement functions of MBP on sow’s immune status postpartum.

Several important hormones and the abilities to digest and absorb nutrients can reflect the recovery state of postpartum sows. Results from the present study showed that the levels of hemoglobin, serum NO, and gastrin, as well as plasma motilin in sows with dietary MBP supplementation, were higher than those in the control diet on days 0 and 7 after delivery. Notably, commercially bred sows may have dropped hemoglobin levels after delivery, causing weakness, fatigue, and poor appetite. Results from one previous study showed that low level of hemoglobin in sows may affect the incidence of stillborn piglets (Normand et al., 2012; Rootwelt et al., 2013). Our data reflect the importance of MBP supplementation diet in increasing postpartum hemoglobin level. Gastrin, a hormone in the stomach, stimulates the secretion of pepsinogen and gastric acid to digest and absorb nutrients in the food (Walsh, 1990). Motilin produced by small intestine is another important hormone for stimulating movement motility of digestive organs (Al-Missri and Jialal, 2021). Therefore, the effects of MBP on postpartum sows in our study may be associated with the improvement of nutrient digestion and absorption function. Furthermore, intestinal absorption of some nutrients or MBP ingredients in sows can increase carbohydrates, disease-fighting proteins, and growth hormones in colostrum and milk. Herein, MBP significantly increased levels of protein, fat, IGF, and EGF (two of the major peptide growth factors) in the colostrum. However, MBP had no effect on these growth factors in the milk on day 7 of lactation. Our results demonstrate that MBP has a different impact on the compositions in colostrum compared with transitional milk.

Milk is composed of proteins, carbohydrates, fat, vitamins, and water, and it is produced from blood and semidigested food. It is reported that all prototype components absorbed into the serum may be the final effective one. Thus, we believed that MBP components absorbed into the milk are considered as main substances of the MBP mixture for improving sows’ and piglets’ health. Herein, a total of 21 components absorbed in the milk were detected. Among them, calycosin, which is mainly from *A. membranaceus* has been reported to increase estrogen receptor expression and upregulation of NO production (Xu et al., 2011). Albiflorin, an active ingredient in Radix Paeoniae Alba, can be exposed at high levels in immune relevant organ or tissues such as the spleen, facilitating immunoregulatory activities (Fei et al., 2016). Ursolic acid can enhance the cellular immune system, regulate microbial diversity in the intestine, and perform antioxidation, anti-inflammation activities (Jang et al., 2009; Wang et al., 2018). Disturbance of the intestinal microbial community by ursolic acid contributes to its function as a regulator of fat deposition. Based on these previous studies, calycosin, albiflorin, and ursolic acid are considered to be highly correlated with the MBP efficacy for sows and piglets.

Except the contribution of metabolites and microbiota to the milk quality, the influences of breast milk on the development of gut microbiota in newborn animals are well-known. To our best knowledge, our findings, rarely reported previously, identified differences according to the food feeding with the MBP in milk microbiota and metabolites. First, we found that lactation time may influence the observed species and Chao1 index, which was consistent with the research of Chen et al. (2018). MBP caused changes in the structure of the microflora in the colostrum and transitional milk. Specifically, the relative abundance of Proteobacteria was increased with MBP supplementation, whereas that of Firmicutes was decreased, especially on day 7 of lactation. Similar results were also obtained by Hou et al., and they hypothesize that the bacteria can be transferred from the rumen to the mammary gland due to the observed similar species in the milk and rumen (Hou et al., 2020). These data indicate the influences of MBP on the local microbiota. In addition, the genus level of NK4A214_group and *Desulfovibrio* was significantly increased in the milk of MBP group on day 7 of lactation. It has been reported that the genera *Ruminococcus* and *Oscillospiraceae* NK4A214 were associated with ewes selected for high milk persistence (Martinez Boggio et al., 2021). In the study by Fan et al., Spearman correlation analysis showed that milk protein content, total volatile fatty acid (VFA), acetate, and propionate concentrations were positively correlated with the relative abundances of the genera *Desulfovibrio* (Fan et al., 2020). Thus, the genus change by MBP administration may be associated with milk production traits in sows. Besides, high levels of *Staphylococcus*, *Corynebacterium*, and *Aerococcus*, which may be related to cow’s mastitis or cause serious losses in dairy products (Oikonomou et al., 2014; Derakhshani et al., 2018), were inhibited by MBP supplement. The metabolome map revealed main pathways such as aminoacyl-tRNA biosynthesis, metabolic pathways, protein digestion and absorption, arginine biosynthesis, and biosynthesis of amino acids based on the milk DFMs between MBP and control. In addition, the three major classes of nutrients (carbohydrates, proteins, and lipids) are transformed into each other through metabolic pathways. In the colostrum of MBP group, some metabolites were upregulated, such as adenosine 5’-monophosphate (AMP) and sarcosine. AMP can increase the amount of intracellular ATP, and sarcosine is involved in forming creatine to provide energy and improve physical performance. Differently, some metabolites upregulated in the transitional milk including essential fatty acids α-linolenic acid (C18:3n-3) and γ-linolenic acid (C18:3N6) fatty acids, critical amino acids L-ornithine, L-aspartic acid, L-citrulline, and L-valine, as well as D-calcium pantothenate. Based on these results, we consider that MBP mainly affects the energy metabolism and metabolic pathway in the colostrum, although it mainly affects the nutrients including fatty acids and protein in the milk collected on the day 7 after delivery. For correlation analyses between bacterial genera and metabolites, bacteria including *C. profftella*, *Sulfurospirillum*, and *Chryseobacterium* may be associated with the metabolism of monounsaturated fatty acid anion. Herein, we focused on the analysis of the effects of MBP on the microflora and metabolites in sow’s milk on days 0 and 7 of lactation (data for 21 days in Supplementary Figure 2) to provide theoretical basis for studying the effect of breast milk containing CHM on the nutrition and immunity of piglets.

## Conclusion

In summary, our findings provide new insights into the beneficial effects of MBP, the active components of the MBP into milk, the microbiota, and metabolomic profile of breast milk from sows fed with MBP. MBP should be considered as a potential diet supplementation for lactating sow.

## Data Availability Statement

The datasets presented in this study can be found in online repositories. The names of the repository/repositories and accession number(s) can be found below: https://www.ncbi.nlm.nih.gov/, PRJNA755976.

## Ethics Statement

The animal study was reviewed and approved by The Experimental Animal Research Ethics Committee of Northeast Agricultural University (SRM-11).

## Author Contributions

JG and YL contributed to the conception and design of the work and drafted the work and substantively revised it. MH substantively revised the manuscript. JG, WJ, JH, and CX performed the research. YZ and BZ analyzed data. All authors read and approved the final manuscript.

## Conflict of Interest

YL was employed by company Harbin Herb & Herd Bio-Technology Co., Ltd., Harbin, China. BZ was employed by Liaoning VICA Agriculture and Animal Husbandry Ecological Food Co., Ltd. JH was employed by Harbin Lvdasheng Animal medicine Manufacture Co., Ltd. The remaining authors declare that the research was conducted in the absence of any commercial or financial relationships that could be construed as a potential conflict of interest. The reviewer BS declared a shared affiliation with several of the authors, JG, WJ, YZ, CX, YL, to the handling editor at the time of review.

## Publisher’s Note

All claims expressed in this article are solely those of the authors and do not necessarily represent those of their affiliated organizations, or those of the publisher, the editors and the reviewers. Any product that may be evaluated in this article, or claim that may be made by its manufacturer, is not guaranteed or endorsed by the publisher.
