# Supplementary material for: Effects of Dietary Modified Bazhen on Reproductive Performance, Immunity, Breast Milk Microbes, and Metabolome Characterization of Sows
Source: Front Microbiol. 2021 Nov 15;12:758224. doi: 10.3389/fmicb.2021.758224 (PMC8634670; doi:10.3389/fmicb.2021.758224)
Supplement: Supplementary file 1 [file Data_Sheet_1.docx]

**Supplementary figure S1 Milk metabolomics analysis of sows.** (A) OPLS-DA score chart of control group (green) and MBP group (red) on days 0 and 7 of lactation. (B) Validation plot obtained from permutation tests. (C) Volcano plot displaying the p-value versus the fold change for each target in a MBP group, relative to the control group on days 0 and 7 postpartum; each point represents a type of metabolite, the green dots in the figure represent the down-regulated differential expression metabolites, the red dots represent the up-regulated differential expression metabolites, and the black dots represent the detected but insignificant metabolites.


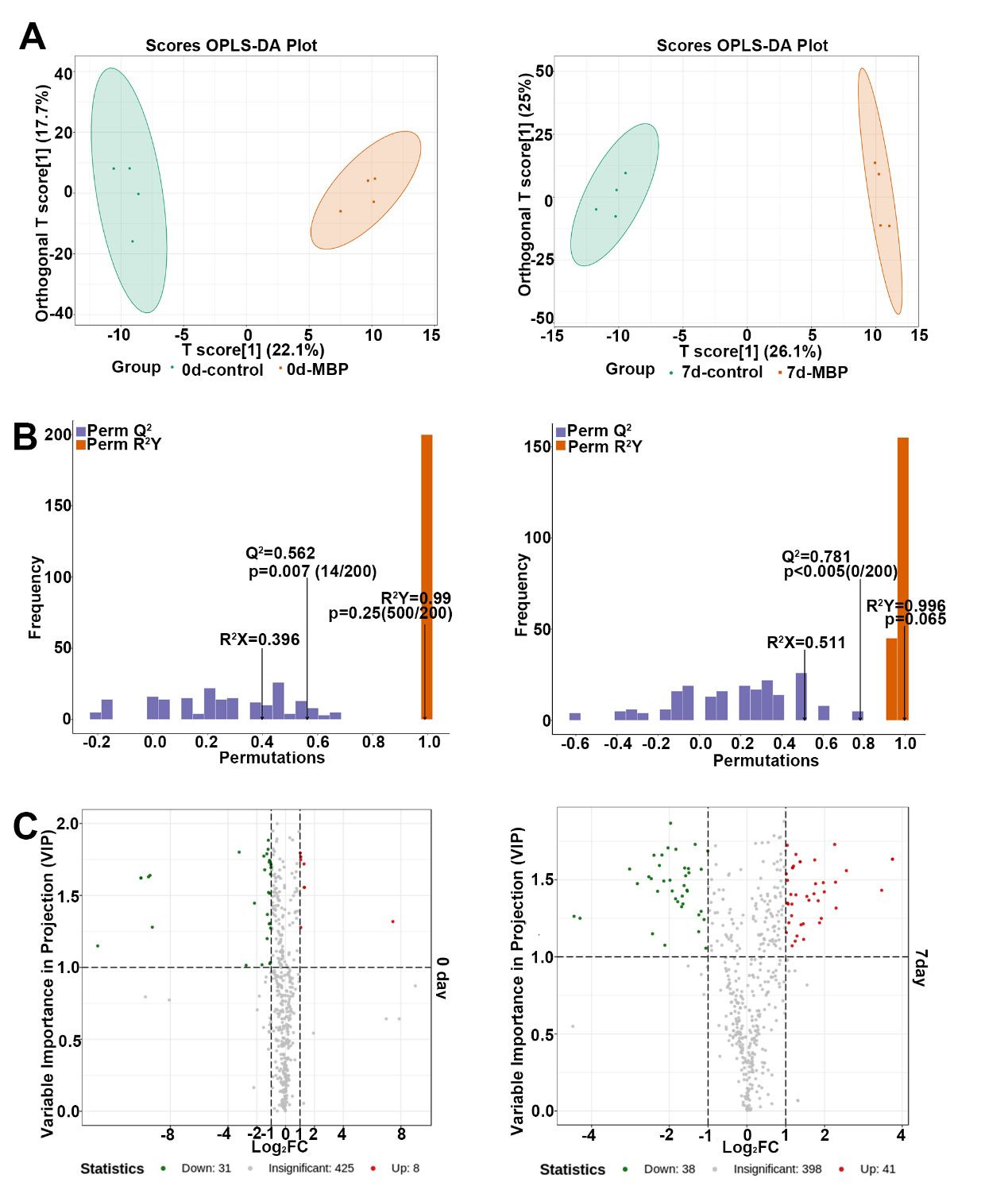


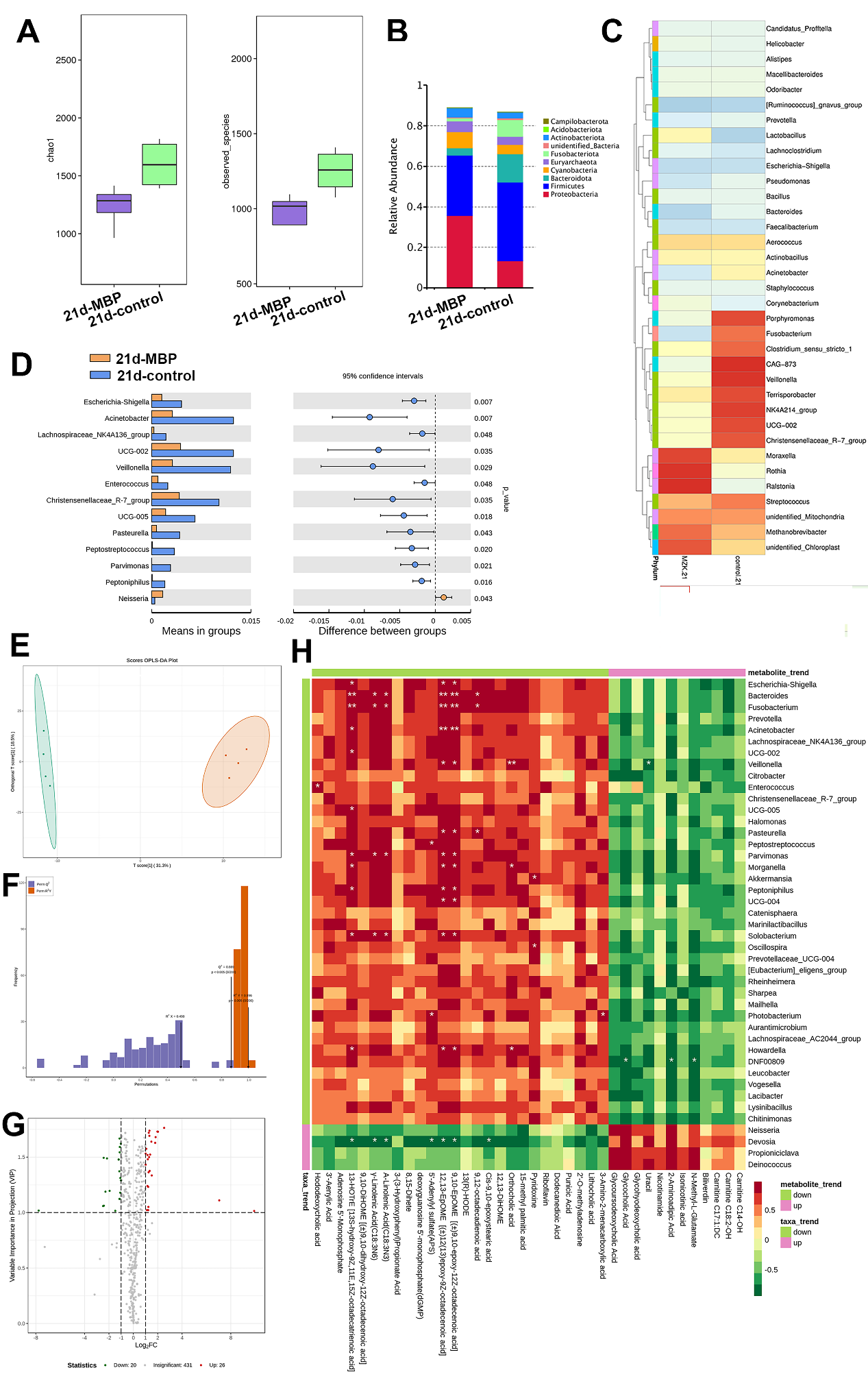


**Supplementary figure S2** Analysis of the effects of modified Bazhen powder (MBP) on the microflora and metabolites in sow’s milk on day 21 of lactation. Microbial alpha‐diversity assessed by (A) Chao1 richness estimator and observed species were calculated. (B) Phylum level. (C) Relative abundance of bacterial taxa at the genus level (top of 35). (D) Significant differences in bacteria at the genus level in milk samples between the two groups. (E) OPLS-DA score chart of control group (green) and MBP group (red). (F) Validation plot obtained from permutation tests. (G) Volcano plot displaying the p-value versus the magnitude of the change. (H) Heat map correlation showing the associations among bacterial genera and metabolite in sow’s milk on day 21 of lactation. p-values for pairwise comparisons of metabolites and bacterial genera. P-values are shown as *P < 0.05 and *P < 0.01.

**Supplementary Table S1** Composition and nutrient content of the basal diet (based on dry matter)

|  | Content (%) | | |
| --- | --- | --- | --- |
| **Ingredient** | Late gestation | Lactation | |
| Corn | 53.65 | | 63.6 |
| Soybean meal, 43% | 10 | | - |
| Soybean meal, 46% | - | | 12.5 |
| Wheat bran | 20 | | 8 |
| Rice bran meal | 10 | | - |
| Dicalcium phosphate | 0.65 | | 0.4 |
| Salt | 0.4 | | 0.4 |
| Sodium bicarbonate | 0.2 | | 0.2 |
| Dicalcium phosphate | - | | 0.4 |
| Limestone | 0.1 | |  |
| Fish meal | - | | 3 |
| fermented soybean meal | - | | 4 |
| Glucose | - | | 1 |
| premix | 5 | | 4 |
| Total | 100 | | 100 |
| **Nutrient composition** |  | |  |
| Digestible energy (MJ·kg−1) | 2905 | | 3320 |
| Crude protein (%) | 14 | | 17.5 |
| Crude fiber | 2.5 | | 2.9 |
| Ash | 7 | | 5 |
| Calcium (%) | 0.86 | | 0.86 |
| L-Lysine | 0.85 | | 1.15 |
| Valine | 0.65 | | 0.88 |

The premix provided the following per kilogram of diets: Gestation: vitamin A, 11,000 IU; vitamin D3, 1,500 IU; vitamin E, 15 IU; vitamin K3, 1.6 mg; vitamin B1,1.5 mg; vitamin B2, 3.0 mg; vitamin B6, 1.5 mg; vitamin B12, 0.015 mg; niacin, 22.5 mg; *D*-pantothenic acid, 15 mg; folic acid, 2.5 mg; biotic,0.2 mg; Fe, 85 mg; Cu, 7.5 mg; Zn, 75 mg; Mn, 35 mg; I, 0.5 mg; Se, 0.3 mg; Lactation: vitamin A, 6,500 IU; vitamin D3, 1,550 IU; vitamin E, 15.5 IU; vitamin K3, 1.6 mg; vitamin B1, 1.6 mg; vitamin B2, 3.1 mg; vitamin B6, 1.5 mg; vitamin B12, 0.015 mg; niacin, 23 mg; *D*-pantothenic acid, 15.5 mg; folic acid, 2.5 mg; biotin, 0.2 mg; Fe, 85 mg; Cu, 10 mg; Zn, 100 mg; Mn, 50 mg; I, 0.5 mg; Se, 0.3 mg.

**Supplementary Table S2**

Identification of significant differential metabolites in the milk from lactating sows.

| Metabolites | VIP | Log2FC | Tendency |
| --- | --- | --- | --- |
| **Colostrum** |  |  |  |
| L-Tryptophan | 1.716904 | -1.0171 | down |
| Sarcosine | 1.318571 | 7.443381 | up |
| 3'-Aenylic Acid | 1.748149 | 1.045123 | up |
| Adenosine 5'-Monophosphate | 1.556064 | 1.297122 | up |
| Inosine 5'-Monophosphate | 1.768294 | 1.050674 | up |
| Tryptamine | 1.71174 | -1.00753 | down |
| 2-Hydroxyisocaproic Acid | 1.36774 | -1.26463 | down |
| 3-Hydroxy-3-Methyl Butyric Acid | 1.149048 | -13.0361 | down |
| Phenyllactate(Pla) | 1.03374 | -1.06236 | down |
| 3-(3-Hydroxyphenyl)Propionate Acid | 1.639626 | -9.409 | down |
| deoxyguanosine 5'-monophosphate(dGMP) | 1.556064 | 1.297122 | up |
| 5'-Adenylyl sulfate(APS) | 1.277211 | 1.049417 | up |
| 12,13-EpOME[(±)12(13)epoxy-9Z-octadecenoic acid] | 1.621415 | -10.0384 | down |
| 9,10-EpOME[(±)9,10-epoxy-12Z-octadecenoic acid] | 1.621415 | -10.0384 | down |
| 3-hydroxyphenylacetic acid | 1.014225 | -2.74333 | down |
| 13(R)-HODE | 1.514437 | -1.10899 | down |
| L-Isoisoleucine | 1.820897 | -1.21398 | down |
| Orthocholic acid | 1.445993 | -2.15908 | down |
| 3-(pyrazol-1-yl) -L-alanine | 1.520859 | -1.18585 | down |
| Mannose 1-phosphate | 1.883728 | -1.19843 | down |
| 4-Hydroxy-3-methylbenzoic acid | 1.629094 | -9.51379 | down |
| L-Tyrosine | 1.731314 | -1.13974 | down |
| L-Histidine | 1.678201 | -1.45004 | down |
| L-Ascorbate | 1.64745 | -1.05024 | down |
| 6-Aminocaproic Acid | 1.742908 | -1.10383 | down |
| DL-Pipecolic Acid | 1.302278 | -1.13983 | down |
| 2'-Deoxycytidine-5'-Monophosphate | 1.279066 | -9.24761 | down |
| Punicic Acid | 1.273212 | -1.0441 | down |
| 5-Aminolevulinate | 1.78893 | -1.30212 | down |
| 5-amino-1-[3,4-dihydroxy-5-(hydroxymethyl)oxolan-2-yl]imidazole-4-carboxamide | 1.718753 | 1.27932 | up |
| DL-Leucine | 1.742908 | -1.10383 | down |
| L-Tryptophanamide | 1.017816 | -1.64101 | down |
| 2'-O-methyladenosine | 1.795082 | 1.014342 | up |
| Lithocholic acid | 1.800961 | -3.2231 | down |
| L-Isoleucine | 1.693622 | -1.02086 | down |
| LysoPC 20:5(2n isomer) | 1.199421 | -1.28111 | down |
| Carnitine C18:2-OH | 1.305797 | -1.07271 | down |
| Carnitine C7:DC | 1.773353 | -1.51143 | down |
| Methylguanidine | 1.025979 | -1.09301 | down |
| **Transitional milk** |  |  |  |
| L-Threonine | 1.481057 | 1.968086 | up |
| Hexanoyl Glycine | 1.697935 | -1.82879 | down |
| Glycoursodeoxycholic Acid | 1.101675 | 1.244061 | up |
| Guanosine Monophosphate | 1.367035 | 1.597252 | up |
| Orotic Acid | 1.22109 | 1.079825 | up |
| 2-Hydroxybutanoic Acid | 1.241978 | -1.10424 | down |
| Phenyllactate(Pla) | 1.208002 | 1.392816 | up |
| 13-HOTrE [13S-hydroxy-9Z,11E,15Z-octadecatrienoic acid] | 1.21415 | 1.451888 | up |
| 9,10-DiHOME [(±)9,10-dihydroxy-12Z-octadecenoic acid] | 1.616835 | 1.371226 | up |
| γ-Linolenic Acid(C18:3N6) | 1.577631 | 1.175267 | up |
| Arachidic Acid(C20:0) | 1.343701 | -1.64661 | down |
| Α-Linolenic Acid(C18:3N3) | 1.577631 | 1.175267 | up |
| N-Acetylmethionine | 1.220949 | 1.871377 | up |
| Ribulose-5-Phosphate | 1.316545 | 2.297433 | up |
| Argininosuccinic acid | 1.363811 | 1.837034 | up |
| D-Malic acid | 1.13512 | 1.290049 | up |
| Tetradecanedioic acid | 1.567994 | -1.17682 | down |
| 2-Hydroxy HippuricAcid | 1.342893 | 1.063631 | up |
| 5'-Adenylyl sulfate(APS) | 1.432046 | 3.470748 | up |
| Hexadecanedioic acid | 1.686499 | -1.00743 | down |
| 12,13-EpOME[(±)12(13)epoxy-9Z-octadecenoic acid] | 1.633502 | 3.74946 | up |
| 9,10-EpOME[(±)9,10-epoxy-12Z-octadecenoic acid] | 1.633502 | 3.74946 | up |
| D-Glucosamine 6-Phosphate | 1.62858 | 1.745745 | up |
| D-Calcium Pantothenate | 1.723911 | 1.038152 | up |
| Iminodiacetic acid | 1.421617 | 1.993234 | up |
| 9,12-octadecadienoic acid | 1.588499 | 1.201642 | up |
| 12,13-DiHOME | 1.616835 | 1.371226 | up |
| D-Mannose 6-phosphate | 1.496414 | 1.045625 | up |
| 3-(pyrazol-1-yl) -L-alanine | 1.40508 | 1.132214 | up |
| L-Ornithine | 1.729062 | 2.260855 | up |
| L-Aspartic Acid | 1.392221 | 1.544466 | up |
| L-Citrulline | 1.113495 | 1.460497 | up |
| L-Valine | 1.538359 | 1.016493 | up |
| 5-Oxoproline | 1.342091 | 1.158993 | up |
| Histamine | 1.249287 | 1.915531 | up |
| L-Asparagine Anhydrous | 1.401958 | 1.2614 | up |
| L-Cystathionine | 1.474121 | 1.769086 | up |
| L-Glutamine | 1.157391 | 1.017181 | up |
| S-Adenosyl-L-Methionine | 1.266829 | 1.151874 | up |
| Uracil | 1.867137 | -1.96499 | down |
| Riboflavin | 1.07166 | 1.165593 | up |
| L-Homoserine | 1.40896 | 1.728519 | up |
| 3-Hydroxyhippuric Acid | 1.347851 | 1.043655 | up |
| DL-Pipecolic Acid | 1.05609 | -1.05673 | down |
| 2'-Deoxycytidine-5'-Monophosphate | 1.706963 | -2.03116 | down |
| 5-amino-1-[3,4-dihydroxy-5-(hydroxymethyl)oxolan-2-yl]imidazole-4-carboxamide | 1.394036 | -1.6667 | down |
| Pterine | 1.497171 | -1.98206 | down |
| Stearidonic Acid | 1.664678 | 1.262774 | up |
| Spermidine | 1.72945 | -1.33148 | down |
| Glycyl-DL-phenylalanine | 1.559166 | 2.561462 | up |
| Dethiobiotin | 1.484367 | 2.283636 | up |
| 4-Hydroxyhippurate | 1.347851 | 1.043655 | up |
| Dodecylcarnitine | 1.462295 | -1.61876 | down |
| Decanoyl L-Carnitine | 1.54551 | -1.49367 | down |
| Lithocholic acid | 1.272437 | -1.23926 | down |
| (±) -Myristylcarnitine | 1.432746 | -1.54326 | down |
| Carnitine C17:1:DC | 1.569381 | -3.02081 | down |
| Carnitine C18:2-OH | 1.491871 | -2.13973 | down |
| Carnitine C18:0 | 1.507055 | -2.46354 | down |
| Carnitine C18:2 | 1.425353 | -2.30064 | down |
| Carnitine C15:DC | 1.519455 | -2.52542 | down |
| Carnitine C16:2 | 1.325054 | -1.68081 | down |
| Carnitine C14-OH | 1.427284 | -1.92357 | down |
| Carnitine C14:2-OH | 1.149781 | -2.43193 | down |
| Carnitine C14:0 | 1.426291 | -1.53498 | down |
| Carnitine C14:1 | 1.474866 | -2.82216 | down |
| Carnitine C14:2 | 1.376612 | -1.83847 | down |
| Carnitine C12:1 | 1.660412 | -2.19003 | down |
| Carnitine C10:0 | 1.572656 | -1.50545 | down |
| Carnitine C8-OH | 1.592838 | -2.2532 | down |
| Carnitine C9:0 | 1.250147 | -4.3 | down |
| Carnitine C7-OH | 1.659022 | -2.39397 | down |
| Carnitine C8:0 | 1.576356 | -1.60344 | down |
| Carnitine C8:1 | 1.295045 | -1.1789 | down |
| Carnitine C9:0 Isomer1 | 1.263356 | -4.45195 | down |
| Carnitine C10:1 Isomer1 | 1.163149 | -1.24247 | down |
| Carnitine C10:0 Isomer1 | 1.525634 | -1.57696 | down |
| Carnitine C14:2 Isomer 1 | 1.357551 | -1.78216 | down |
| Carnitine C16:2-OH | 1.075253 | -2.11423 | down |
